# Beyond Hepatocellular Carcinoma and Colorectal Metastasis: The Expanding Applications of Radioembolization

**DOI:** 10.3389/fonc.2014.00150

**Published:** 2014-06-16

**Authors:** Omar Zurkiya, Suvranu Ganguli

**Affiliations:** ^1^Division of Interventional Radiology, Department of Radiology, Massachusetts General Hospital, Harvard Medical School, Boston, MA, USA

**Keywords:** radioembolization, neuroendocrine radioembolization, breast radioembolization, melanoma radioembolization, metastatic liver radioembolization

## Abstract

As a relatively safe outpatient procedure, radioembolization can potentially be used to treat any type of tumor within the liver, primary or metastatic. The safety and effectiveness of radioembolization in the treatment of hepatocellular carcinoma (HCC) and metastatic colorectal cancer (mCRC) has led many groups to explore its application in other malignancies. Moreover, other organs, such as the lungs and kidneys, have been explored as targets for therapy. Although the most data for radioembolization is related to HCC and mCRC, there is increasing experience and data regarding metastatic disease to the liver for other primary tumors. We review the current state of liver-directed therapy with radioembolization outside of HCC and mCRC, including metastatic neuroendocrine, breast, and melanoma, as well as limited experiences with other primary malignancies. Applications of radioembolization related to these other cancers and new trends and future directions will be discussed. With increasing use and availability of radioembolization, it promises to serve an expanding role in the repertoire of tools available for treating and managing oncologic disease.

## Introduction

As a relatively safe outpatient procedure, radioembolization can potentially be used to treat any type of tumor within the liver, primary or metastatic. The safety and effectiveness of radioembolization in the treatment of hepatocellular carcinoma (HCC) and metastatic colorectal cancer (mCRC) has led many groups to explore its application in other malignancies. Moreover, other organs, such as the lungs and kidneys, have been explored as targets for therapy. Although the most data for radioembolization is related to HCC and mCRC, there is increasing experience and data regarding metastatic disease to the liver for other primary tumors. We review the current state of liver-directed therapy with radioembolization outside of HCC and mCRC, including metastatic neuroendocrine, breast, and melanoma, as well as limited experiences with other primary malignancies. Applications of radioembolization related to these other cancers and new trends and future directions will be discussed. With increasing use and availability of radioembolization, it promises to serve an expanding role in the repertoire of tools available for treating and managing oncologic disease.

## Radioembolization in Liver Metastases Other than mCRC

Radioembolization has a proven role in the treatment liver metastases from mCRC, however, increasing evidence shows its efficacy in several other primary tumor types. Liver tumors derive relatively more of their blood supply from the hepatic artery than adjacent normal liver, which is primarily perfused by the portal vein. As such, radioembolization may have the potential to treatment a wide variety of liver metastases.

Due to the exploratory nature of treatment of liver metastases beyond mCRC, many studies employ radioembolization only after failing standard treatment or in cases where certain criteria for standard treatment are not met. Large or multiple lesions may preclude surgical resection, with radioembolization offered as a means to control disease progression. In patients who have failed systemic therapies or cannot tolerate further chemotherapy, radioembolization may be offered as a salvage therapy. As such, randomized trials of radioembolization vs. standard therapy are lacking. Nevertheless, several published series of radioembolization of hepatic metastases have shown promising results. Here, we discuss the current state of radioembolization for liver metastases beyond mCRC.

### Neuroendocrine tumor metastases

As a whole, neuroendocrine tumors (NETs) are rare, comprising approximately 0.5% of malignancies. They are often indolent, presenting with symptoms due to hormonal activity or as a result of invasion of local structure from either the primary tumor or metastases. Forty to ninety percent of patients with NETs will have liver metastases at the time of diagnosis (1). These patients have a significantly worse prognosis than those without liver involvement having a 5-year survival of 0–20%.

Surgical resection remains the definitive treatment for NET metastasis. However, due to their often indolent course, patients often present with large and/or multiple lesions, precluding surgical resection. For this reason and in patients who are otherwise not surgical candidates, other forms of therapy are being investigated. NETs are highly vascular, with a great portion of blood supply from the hepatic artery as compared to the adjacent normal liver tissue, which is pre-dominantly supplied by the portal vein. As a result, intra-arterial therapies are being investigated, including radioembolization with Y-90 microspheres.

However, limitations in performing a retrospective review of radioembolization in metastatic NET arise from the heterogeneous methods employed in the various published small-scale series. While large scale, controlled trials of transarterial chemoembolization (TACE) have been performed, radioembolization remains a newer approach and is often performed in patients who have otherwise failed or have contraindications to frontline therapies. Many patients have had previous liver resections or are being treated with various neoadjuvant medications, which may affect response and potential complications. Even the criteria utilized for measuring response varies between series, limiting comparison, and pooling of data. Brown et al. have suggested a framework for reporting of transcatheter treatment of hepatic malignancies (2), although in the absence of widespread adoption of such standards, data from smaller case series cannot be readily aggregated, limiting the ability to draw effective conclusions.

In order to provide a perspective on efficacy, comparison of radioembolization to other intra-arterial treatment modalities such as TACE is useful. Yang et al. reviewed several publications reporting NET treatment with radioembolization. In their study, radioembolization patients had an objective response of 63.1% compared to 58.4% of those treated with TACE. The clinical response rates were 85 and 88.5% for radioembolization and TACE, respectively (1). One and 5-year survival rates for radioembolization patients were 84.7 and 50.5%, while TACE patients were 75 and 30.5%, respectively.

King et al. reported a series of 34 patients treated for NET metastasis with 22 patients (65%) experiencing complete response (CR), partial response (PR), or stable disease (SD) by RECIST criteria (3). One patient died within 1 month and the remaining 11 patients showed progressive disease by imaging. The mean survival was 27.6 ± 2.3 months. Fourteen patients (41%) died from progressive metastatic disease at 1–28 months with mean survival of 14.6 ± 2.2 months, and 20 patients remained alive at the end of the study period with mean survival of 36.7 ± 1.8 months.

Murthy et al. also reported that radioembolization for NET metastasis can be performed after previous TACE with a median survival time of 14 months after radioembolization (4). In a multicenter phase II study with 42 patients of hepatic NET metastases, Rhee et al. investigated the efficacy and safety of radioembolization. They observed that 92 and 94% of patients treated with glass (TheraSpheres, Nordion Inc.) and resin-microspheres (SIR-spheres, Sirtex Medical Limited), respectively showed either PR or SD at 6 months, and median survival was 22 and 28 months, respectively. Though there remains debate about the use of glass vs. resin spheres, their study found no significant difference in survival outcomes (5).

To date, there have been no published controlled trials comparing radioembolization to TACE for metastatic NET. Such trials would provide stronger evidence for providers to choose radioembolization for metastatic NET. However, current data shows promise for the modality. Radioembolization may allow control and possible downstaging of liver metastases, possibly making patients eligible for other treatment options such as radiofrequency ablation, liver resection, or liver transplantation.

### Breast cancer metastases

Breast cancer is the most common malignancy in females, with an estimated lifetime risk of 10–15%. While the overall 5-year survival is >90% when there is only local occurrence, survival in patients with metastatic disease is only 3–20% depending on age and comorbidities (6). Despite advances in treatment, metastatic breast cancer remains a major cause of morbidity and mortality.

Breast cancer most frequently metastasizes to the skeleton, liver, lungs, and brain. Among patients with metastases, approximately 5–20% have liver metastases, although autopsy studies suggest the incidence may be as high as 61% (7). The prognosis of patients with liver metastases is poor with a median survival time in the range of 1–20 months. As with other liver malignancies, surgical resection is considered the only option for cure of breast cancer liver metastases. Unfortunately, up to 57% of breast cancer patients with liver disease are found to have disseminated metastases, precluding surgical intervention (7).

Radioembolization has been explored as a treatment option for breast cancer metastases, and several studies have been published describing outcomes. It is usually offered as a salvage treatment in patients with end-stage disease, history of several previous systemic therapies, and high incidence of extra-hepatic metastases. Nevertheless disease control rates have been reported as high as 70–96% (8, 9). Saxena et al. reported a median survival of 13.6 months after the first radioembolization treatment. While no control group is available from this study, Wyld et al. reported an overall survival of only 4.2 months (10) within a total of 145 studied patients with liver metastases from breast cancer treated with systemic chemotherapy or best supportive care.

Interestingly, radioembolization survival was found to improve with three identified prognostic factors: extent of replacement of hepatic parenchyma by tumor (less involvement associated with increased survival), chemotherapy after radioembolization (yes vs. no; *p* = 0.015), and radiological response to treatment (per RECIST criteria CR/PR vs. SD vs. PD). This suggests that patients who undergo radioembolization may benefit from further chemotherapy. For many patients, the side effects of chemotherapy are difficult to overcome, and given the relative tolerance of radioembolization, a treatment plan involving the use of radioembolization during a “chemotherapy holiday” can be an attractive option.

The studies included in this review were again retrospective case series. The lack of comparative, prospective studies limits any definitive conclusions on the efficacy of this treatment for this population. As opposed to other applications, such as colorectal cancer, which primarily metastasize to the liver, breast cancer often metastasizes beyond the liver. In such patients, radioembolization may not lead to a survival benefit, even if the hepatic disease is controlled. Further studies, including combinations of radioembolization therapy with systemic therapy will be necessary to fully define the role of radioembolization in metastatic breast cancer.

### Uveal melanoma metastases

Uveal melanoma (UM) is the most common primary intraocular tumor in adults and has a pre-dilection to metastasize to the liver. These are often widespread with a poor prognosis, and liver failure is the primary cause of death in these patients. Given the extent of disease in this group of patients, surgical resection and other liver-directed therapies such as thermal ablation are usually not an option.

The first report of metastatic ocular melanoma treatment came from Kennedy et al. (11) who reported a retrospective review of 11 patients (6 females, 5 males) receiving 12 treatments. The duration of time from original diagnosis to radioembolization was a median of 25.5 months (1–118 months). All patients had bi-lobar disease and more than four lesions. Three patients died from extra-hepatic metastases at 2.5, 3, and 18 months post-treatment. One patient was lost to follow-up. Of the remaining eight patients, one developed new hepatic lesions at 14 months and was retreated. The median survival was not reached at end of the study period; however survival at 1 year was 80%. This compares with a report of the Collaborative Ocular Melanoma Study (CMOS) Group finding a median time from diagnosis of metastasis to death of <6 months and a death rate of 80% at 1 year and 92% at 2 years following report of melanoma metastasis (12).

Gonsalves et al. reported a series of 32 patients who underwent radioembolization therapy for UM hepatic metastasis (13). The median overall survival was 10.0 months, and progression-free survival of hepatic metastasis was 4.7 months. Patients were grouped by pre-treatment tumor burden into three categories: <25% (*n* = 25), 25–50% (*n* = 5), and >50% (*n* = 2). Patients with tumor burden <25% had longer median overall survival (10.5 vs. 3.9 months, *p* = 0.0003) and progression-free survival (6.4 vs. 3.0 months, *p* = 0.03) than patients who had a pre-treatment tumor burden of 25% or greater. Patients with CR (*n* = 1), PR (*n* = 1), or SD (*n* = 18) had longer median overall survival (14.7 vs. 4.9 months, *p* = 0.0006) and progression-free survival (7.9 vs. 3.1 months, *p* < 0.0001) than patients with tumor progression (*n* = 12). Similar to other applications of radioembolization, self-limiting grade 1–2 systemic toxicities were the most frequent including fatigue (*n* = 9), indigestion (*n* = 2), and abdominal discomfort (*n* = 5). Grade 3–4 hepatic toxicities were attributed to tumor progression.

Dhanasekaran et al. reported 12 patients with melanoma, who failed systemic therapy and were treated with Y-90 radioembolization (SIR-Spheres) (14). Overall median survival from the diagnosis of liver metastases was 13.9 months (6.2–21.6). Median survival in patients treated with Y-90 radioembolization was 15.5 months. The duration of SD by RECIST criteria in patients treated with Y-90 was found to be 10.10 months with a positive correlation between progression-free duration and survival (*p* = 0.05).

Klingenstein et al. reported results from 13 patients. In their series, pre-treatment hepatic tumor load varied with the majority having tumor load of 25–50% (*n* = 8) and all were confirmed to be FDG avid on PET (15). Treatment response after radioembolization was partial in eight patients (62%), stable in two patients (15%), and progressive in three patients (23%) by RECIST criteria. After 6 months, of the eight patients still alive, two patients showed ongoing PR, four patients had SD, and two had progressive disease.

By PET, the response was less positive with only three patients (23%) showing PR and seven (54%) with progressive disease. Median survival time after radioembolization was 7 months. Patient median survival after diagnosis of metastases was 19 months (range 4–56). Of note, evaluation method (RECIST vs. PET criteria) did not show any significant difference in predicting survival.

### Other primary tumors

Several investigators have reported experiences in treating liver metastases from other primary malignancies. The majority of these are patients with unresectable tumors, chosen on a case by case basis. Aggregate data on response by RECIST criteria using either CT, MRI, or by PET suggests promising results. Stuart et al. reported a cohort of 30 patients with chemo-resistant liver metastases including sarcoma (*n* = 3), esophageal (*n* = 2), endometrial (*n* = 1), lung (*n* = 1), ovarian (*n* = 1), SCC of the anus (*n* = 1), and unknown primary (*n* = 1) as well as colorectal (*n* = 13) and breast cancer (*n* = 7) (16). The 10 patients with liver metastases from other than mCRC and breast cancer reached a median survival of 638 days. The patients who experienced a response or SD after radioembolization went on to have an increased survival in comparison to those with progressive disease (604 vs. 251 days; *p* = 0.001).

Sato et al. reported a cohort of 131 patients including adrenal, angiosarcoma, bladder, cervical, duodenal, esophageal, gastric, lung, lymphoma, ovary, pancreas, parotid, and thyroid cancer, in addition to the previously discussed primary carcinomas (17). In their cohort, 90% of patients experienced either a response or SD by RECIST, an overall rate similar to those reported for more commonly treated HCC and colorectal metastases.

These experiences suggest that radioembolization may have application in nearly any liver lesion, whether primary or metastatic disease. It will take time and experience to accumulate data on each individual tumor type, but as user experience increases, radioembolization may become a standard offer in patients with unresectable hepatic disease that is unresponsive to traditional treatments.

### Complications

Radioembolization remains well-tolerated. Post-embolization syndrome symptoms are common, reported at 67 and 100% (18, 19) by common terminology criteria for adverse events (CTCAE). Though these symptoms were common, only 18% needed hospitalization for one night for pain control or dehydration. These symptoms are typically managed conservatively and generally resolve within 1 week. Grade 3 toxicities of nausea, vomiting, and pain by CTCAE occurred in <20% of patients in the above series with no deaths attributed to the procedure.

The incidence of radioembolization-induced hepatic failure, though a valid concern, is minimal, reported as low as 0–4% (1). The rate of major complications for both radioembolization and chemoembolization are similar, with one review of metastatic NET compiling 423 patients reporting 0.95% of patient dying within 30 days of radioembolization. This was secondary to various reasons including tumor progression as well as one patient from hepatic failure (1). In similar studies with TACE, 3.2% died within 30 days. The most frequent cause, accounting for approximately one-third of these deaths, was hepatic failure due to high tumor burden resulting in hepatic infarction.

As with radioembolization in HCC and mCRC, absolute contraindications to treatment include significant hepatopulmonary shunting and reflux into arteries that supply the gastroduodenal artery region, which may result in non-target radiation. This can lead to gastric or duodenal ulcers, pancreatitis, cholecystitis, or radiation pneumonitis and emphasizes the importance of the pre-SIRT procedure in which non-target vessels are embolized prior to the treatment. Radioembolization-induced hepatic failure, though rare, remains a concern and may be avoided by treating one lobe at a time and proper patient selection.

## Radioembolization Outside the Liver

There is interest in using radioembolization outside the traditional treatment bed of the liver to treat tumors. However, the data is sparse, and limited to case reports. Therefore, aside from safety profiles, little conclusions can be made in this realm.

Hamoui et al. describe a case of a patient with a renal mass who was referred for palliative therapy because of advanced stage of disease (20). Biopsy had revealed renal cell carcinoma of an aggressive sarcomatoid subtype. Due to advanced age, systemic chemotherapy was not an option. The patient was also found to have liver metastases. The patient subsequently underwent planning mesenteric and renal angiography and returned 1 week later for radioembolization of the renal tumor. The authors gave intravenous hydration and admitted the patient for observation. She reported only mild nausea and pain after the procedure, which were medically controlled. At 8 weeks, CT showed new areas of necrosis in the renal tumor, which was itself stable in size. The renal lesion remained stable at 9 months; however was found to have new pulmonary nodules. The patient died 23 months after radioembolization. This compares favorably to the expected survival of sarcomatoid renal cell carcinoma in the range of 3–10 months. In carefully selected patients, radioembolization may be a viable option in advanced or metastatic renal cell carcinoma.

Despite public efforts, lung cancer remains a major cause of cancer related death worldwide. In addition to primary tumors, metastatic disease to the lung is common. Surgical resection remains the definitive treatment and thermal ablation is also becoming a well-established approach. However, these have limited roles when there are greater than three lesions or if the lesion is >3 cm. Localized transarterial treatment of lung tumors, with delivery via the bronchial arteries, is therefore of great interest.

Ricke et al. described Y-90 resin-microspheres treatment to the lungs in two patients with diffuse metastatic disease of colorectal and renal cell cancer, respectively (21). The decision was made to attempt radioembolization in these patients in only a portion of the lung, with the intent of preserving untreated areas of lung in case of radiation-induced pneumonitis. Lung function tests remained normal through 4 weeks in both patients and no evidence of pneumonitis was seen on CT up to 12 weeks in one patient. Targeted nodules were either stable or showed partial remission, whereas non-targeted nodules showed progressive disease. The patients, whose disease burden was high before undergoing the treatment, died at 6 and 9 months, respectively.

Pneumonitis is one of the primary concerns limiting radioembolization in patients with high lung shunt fraction. Interestingly, neither of the patients in this report demonstrated radiation-induced lung injury. However, pulmonary function tests were only carried out to 4 weeks, and radiation effects may take months to years to develop. Nevertheless, in patients with pulmonary lesions who have failed traditional therapies, radioembolization may offer a chance of increased survival.

## Conclusion

As experience with Y-90 radioembolization grows, there is increasing interest in exploring new applications as reviewed here. These studies increase the already extensive data supporting the safety and efficacy of Y-90 radioembolization in various applications. Head to head, comparative studies are still needed to fully define the role Y-90 in these emerging uses. The collective experiences described here and elsewhere suggest a potentially wide ranging and significant role of Y-90 radioembolization in oncology that is yet to be realized.

## Conflict of Interest Statement

The Guest Associate Editor Cicero Matthew R. Habito declares that, despite being affiliated to the same institution as authors Omar Zurkiya and Suvranu Ganguli, the review process was handled objectively and no conflict of interest exists. The authors declare that the research was conducted in the absence of any commercial or financial relationships that could be construed as a potential conflict of interest.
